# Activation and Genetic Modification of Human Monocyte-Derived Dendritic Cells using Attenuated *Salmonella typhimurium*

**DOI:** 10.1100/tsw.2010.37

**Published:** 2010-03-05

**Authors:** Agnieszka Michael, Justin John, Brendan Meyer, Hardev Pandha

**Affiliations:** ^1^ Postgraduate Medical School – Oncology, University of Surrey, Manor Park, Guildford, UK; ^2^ Oncology Department, St George's University of London, United Kingdom

**Keywords:** salmonella, dendritic cells, cytokines, gene transfer

## Abstract

Live attenuated bacterial vectors, such as *Salmonella typhimurium*, have shown promise as delivery vehicles for DNA. We have examined two new strains of *S. typhimurium* and their impact on dendritic cell maturation (CD12-*sifA/aroC* mutant and WT05-*ssaV/aroC*, both in TML background). Strain WT05 matured dendritic cells in a more efficient way; caused higher release of cytokines TNF-α, IL-12, IL-1β; and was efficient for gene transfer. These findings suggest that the genetic background of the attenuation can influence the pattern of inflammatory immune response to *Salmonella* infection.

### INTRODUCTION

Dendritic cells (DC) are the most potent antigen-presenting cells (APC) and have their roles in the regulation of host innate and adaptive immune responses. Their functional characteristics depend on their state of maturation. Once the antigens, such as bacteria, are captured by immature DC, the cells begin the maturation process associated with rapid decline of their ability to capture new antigens; rise in the expression of MHC class I, class II, costimulatory, and other surface molecules; and enhanced cytokine and chemokine production as reviewed in Banchereau and Steinman[1]. Depending on the maturation signal, DC activate a variety of T cells that differentiate further into Th1- or Th2-directed immune response.

*Salmonella typhimurium* is an intracellular pathogen that can cause severe infection in man. Live attenuated bacterial vectors, such as *S. typhimurium*, have shown considerable promise as efficient delivery vehicles for DNA. The potential advantage of such bacterial vectors is their ability to transfer efficiently foreign DNA under eukaryotic promoter control to key inductive cells of the immune system, such as DC, and to simultaneously up-regulate the expression of appropriate costimulatory molecules, such as B7.1 and B7.2. The transferred DNA is then subsequently expressed by host cells leading to B- and T-cell stimulation, and induction of a protective immune response as reviewed in Chatfield et al.[2]. Recognition of bacteria by the innate immune system, mediated by the toll-like receptors-4 (TLR-4), also leads to cytokine production by DC and natural killer cells (NK) that shape the type of response to infection. DC have also been recently implicated in mediating bacterial transport across the epithelial surface[3].

The interaction between DC and *Salmonella* is therefore of particular importance in generating a desirable immune response as well as in gene transfer process, and is dependent on both DC activation and maturation, as well as the cytokines produced.

*S. typhimurium* is known to mature DC. However, various attenuated strains appear to have differing effects, with certain strains inducing more efficient maturation and therefore showing a potential advantage as gene transfer vehicles[4,5,6]. In addition, the pattern of cytokine production includes both Th1 and Th2 cytokines, and may be triggered by virulence factors[4].

We have examined two new attenuated strains of *S. typhimurium* TML and their impact on DC maturation. The WT05 strain contains defined (independently attenuating) deletions in two genes, *aroC* and *ssaV*, and the CD12 strain contains a defined deletion in the *aroC* gene, as well as a second defined deletion in the s*ifA* gene. The *aroC* gene encodes chorismate synthase, which is required for the biosynthesis of aromatic compounds. The *ssaV* gene is encoded in the SPI-2 region and is a structural gene that constitutes part of the secretion apparatus encoded by this region. The *sifA* gene is involved in maintaining the *Salmonella*-containing vacuoles (SCV) and mutation results in *Salmonella* invading the host cytosol, instead of remaining within the SVC.

We compared the cytokine profile following infection of DC by CD12 and WT05, and compared it with the background strain TML, a wild type of *Salmonella*. We have also shown that both strains are capable of gene transfer to DC *in vitro* using EGFP (enhanced green flourescent protein) as a reporter gene.

### MATERIALS AND METHODS

#### Culture of DC from Peripheral Blood Monocytes

Human DC were cultured from peripheral blood mononuclear cells (PBMC) as per standard protocol. Briefly, PBMC were obtained from healthy donors' buffy coat FICOLL-Histopaque (Sigma-Aldrich). PBMC were resuspended in growth medium (RPMI-1640 containing 10% heat-inactivated fetal calf serum [FCS], 50 U/ml penicillin, and 50 µg/ml streptomycin, all purchased from Sigma-Aldrich) at a final concentration of $3 \times 10^6$ cells/ml. Cells were incubated in 100-mm plastic Petri dishes for 2 h at 37°C. Nonadherent cells were removed by pipetting and the remaining adherent cells were cultured in DC medium consisting of growth medium supplemented with 100 ng/ml recombinant human GMCSF (granulocyte-macrophage colony stimulating factor) (Leucomax, Sandoz) and 50 ng/ml recombinant human IL-4 (interleukin-4) (Peprotech EC). Fresh cytokines were added every 2–3 days up to day 7.

#### Bacterial Strains and Growth Conditions

The bacterial strains used in this study are CD12-*sifA/aroC* mutant in a TML background and WT05-*ssaV/aroC* mutant in a TML background (obtained from Microscience). Bacteria were grown in Luria-Bertani (LB) medium (Sigma) supplemented with a mixture of 4-aminobenzoic acid (BDH), 2,3-dihydroxybenzoic acid (Sigma), L-phenylalanine, L-tryptophan, and L-tyrosine (Sigma) (Aromix).

#### Infection of DC with *Salmonella*

The bacteria were grown in LB broth supplemented with Aromix at 37ºC with moderate shaking. Immediately prior to infection, cells were washed with saline and suspended in complete medium without antibiotic or serum. The bacteria were added at different ratios for 30 min. Following the infection, the medium was supplemented with gentamycin (100 µg/ml) (Gibco BRL) for 60 min, and then replaced with fresh medium containing 10% serum, 2 m*M* glutamine, and gentamycin (16 µg/ml). The growth factors

GMCSF and IL-4 were then added, and DC were incubated for 24–48 h. The polymyxin B (Sigma-Aldrich) was added at 25 µl/ml and 50 µl/ml of culture.

### Assessment of Gene Transfer from *S. typhimurium* to Human Monocyte-Derived DC

Gene transfer from *S. typhimurium* to human monocyte-derived DC was assessed using EGFP plasmid. The EGFP was cloned into pIRES2 plasmid with eukaryotic expression obtained from Clontech Labs. An attenuated strain of *S. typhimurium* harbouring EGFP plasmid was added to DC at different MOI (multiplicity of infection ratio) that varied from 50:1 to 1:1 and incubated for 30 min at 37ºC. Cells were then incubated with medium containing gentamycin (100 µg/ml) for 1 h. After 1 h, the medium was changed to complete medium with 10% serum, 2 m*M* glutamine, and gentamycin (16 µg/ml). After another 4 h of incubation, 10 µg/ml of tetracycline was added to block intracellular bacterial multiplication and, thereafter, incubation was continued for another 48 h. The medium was then removed and the cells were washed and evaluated by fluorescent microscopy.

### Analysis of Maturation Markers

Maturation of DC was determined 24 and 48 h after infection with *S. typhimurium* or after 24-h exposure to lipopolysaccharide (LPS) from *S. typhimurium* (Sigma-Aldrich) by analysis of the expression of the following surface markers: MHC class I, class II, CD83, CD86, and CD40. The harvested DC were labelled with the relevant fluorescein isothiocyanate (FITC)- or phycoerythrin (PE)-conjugated antibody at a concentration of $1–5 \times 10^5$ cells for 30 min on ice. Additional samples were labelled with an isotype-matched irrelevant antibody to account for nonspecific binding. The cells were then washed twice in 2 ml of wash buffer (1,500 rpm, 5 min, 4$^o$C) and finally resuspended in 250 µl of 1% paraformaldehyde in phosphate buffered saline (PBS). All the samples were then analysed in a flow cytometer (Beckton Dickinson). Additionally treated DC were analysed for expression of mannose receptor by staining with relevant antibody.

### Cytokine and Chemokine Analysis

All 48-h cultures of DC were analysed for cytokine secretion, including IL-12, IL-12 p70, TNF-α, IL-10, and IL-1β using the Cytometric Bead Array kit (BD Biosciences). Briefly, 50 µl of mixed capture beads were added to the appropriate assay tubes, mixed with the supernatants, and incubated for 3 h. Samples were then analysed on the flow cytometer together with the appropriate standards and PE detection reagents. The chemokine secretion CCL3, CCL4, and CCL5 was analysed using standard ELISA method. All experiments were conducted at least twice.

## RESULTS

### Infection of DC with *S. typhimurium* Strain CD12 and WT05

We evaluated the *in vitro* pattern of infection of human DC with CD12 and WT05 strains. The different infection ratio (MOI) was tested as *Salmonella* infection was very toxic to the DC. The MOI of 50:1 killed 90% of the cells within 24 h. The MOI of 2:1 and 1:1 enabled us to retrieve 50% of live DC as estimated by trypan blue viability count. The ability of CD12 and WT05 strains to mature DC was evaluated after DC infection. The expression of maturation markers MHC I, MHC II, CD83, CD86, and

CD40 after infection of DC with two novel strains was subsequently compared with the maturation pattern induced by wild-type *S. typhimurium* LPS. Both bacterial strains and LPS were capable of maturing DC, but there were marked differences between the level of expression of maturation markers, with strain WT05 being clearly more active. The mean channel fluorescence (MCF) levels of CD83 expression induced by WT05 was 76.8% as compared with 53.2% for strain CD12, and the CD86 expression level was 87.9 vs. 73.7% for WT05 and CD12, respectively (Fig. 1).

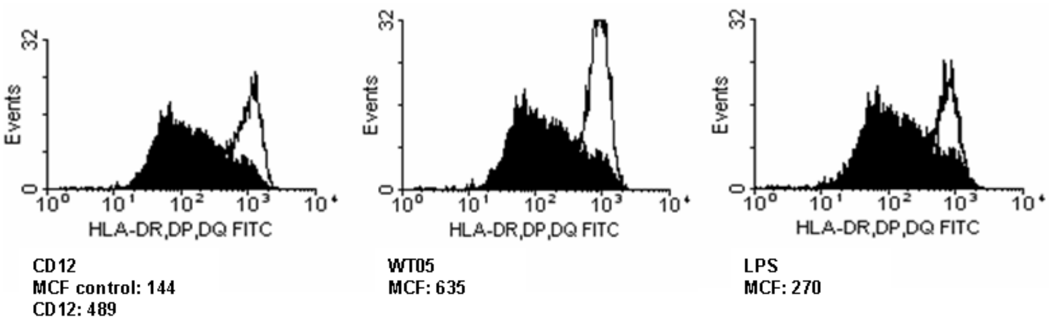

**A**

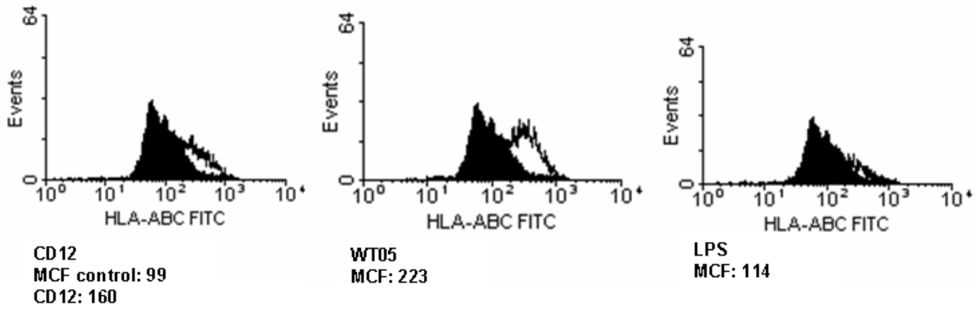

**B**

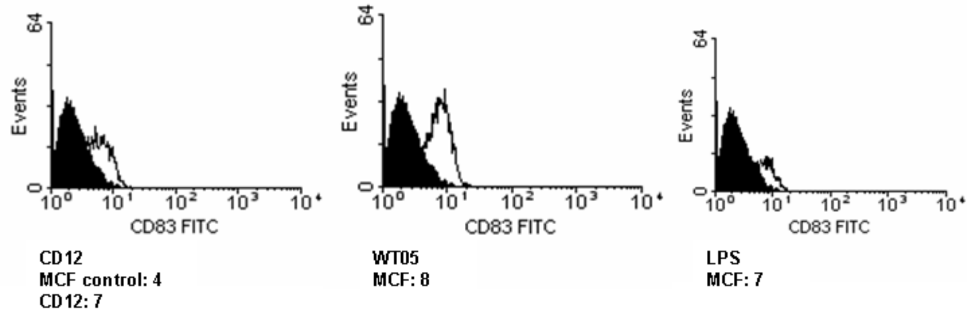

**C**

**FIGURE 1.** Expression of maturation markers on DC infected with *S. typhimurium*. Graphs show FACS staining data and expression of MHC II (HLA-DR, DP, DQ) (A), MHC I (HLA-ABC) (B), CD83 (C), CD86 (D), CD40 (E) of control (uninfected, black filled-in graphs) DC; DC matured with *S. typhimurium* strain CD12 (black line, empty graph); DC matured with *S. typhimurium* WT05 (black line, empty graph); DC matured with *S. typhimurium* wild-type LPS (black line, empty graph), respectively. MCF is marked under each graph. Infection of DC with strain WT05 resulted in higher expression of maturation markers than strain CD12 or exposure to *S. typhimurium* LPS. (Analysed on the WinMDI software.)

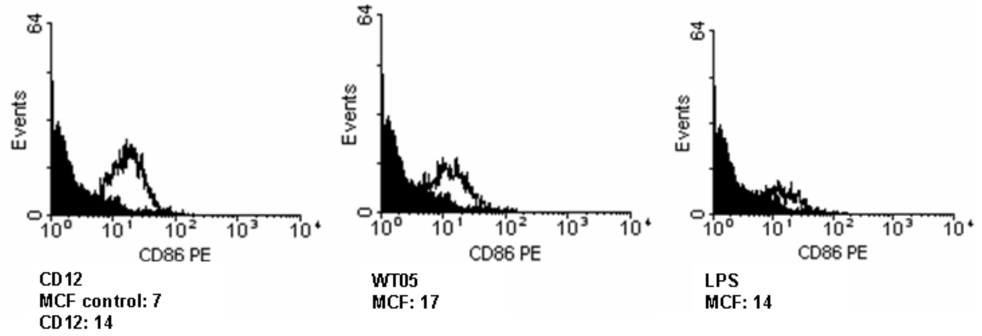

**D**

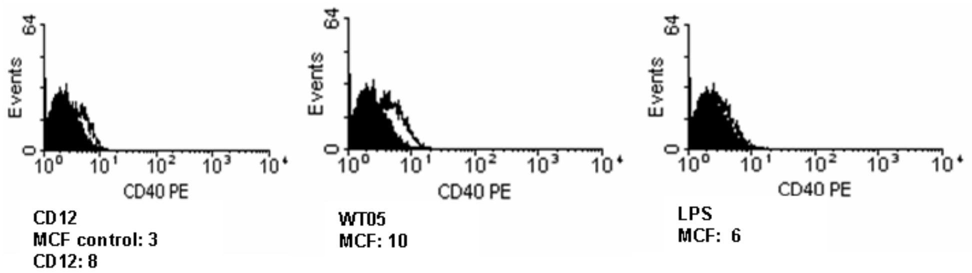

**E**

**FIGURE 1 continued.**

### Cytokine and Chemokine Secretion by DC in Response to Infection with CD12 and WT05

DC maturation can also be characterised by cytokine production, which is closely associated with activation of T cells. Depending on the maturation stimulus, DC can either activate CD8 T cells or CD4-expressing T-helper cells. DC matured by pathogens synthesise high levels of IL-12 and TNF-α[7,8]. The levels of IL-12 p70, TNF-α, IL-10, and IL-1β were measured following infection with CD12, WT05, and the background wild type to both strains - *S. typhimurium* strain TML. The levels of IL-12 p70 were significantly higher (*p* value 0.05) for the WT05 strain than for the CD12 strain and resembled the levels induced by the wild-type strain. Secretion of TNF-α was also higher for DC matured by the WT05 strain as compared to the CD12 and wild-type S. *typhimurium*, but did not reach statistically significant difference. IL-10 production and IL-1β secretion followed the same pattern without reaching the statistical significance (*p* value 0.40) (Fig. 2).

To evaluate the impact of infection of DC with attenuated strains of *S. typhimurium*, the 48-h supernatants of infected DC were tested for the presence of CCL3, CCL4, and CCL5. The supernatants contained high levels of all three of the tested chemokines produced by DC infected with *S. typhimurium* strains CD12, WT05, and TML, as well as DC exposed to *S. typhimurium* LPS; there was no difference between the bacterial strains (data not shown).

### Assessment of Gene Transfer using CD12 and WT05 Strains in Human DC

Evidence of gene transfer using attenuated *Salmonella* under eukaryotic control has been shown in a variety of different tumour models[9,10,11,12]. We evaluated both strains CD12 and WT05 as vectors for delivery

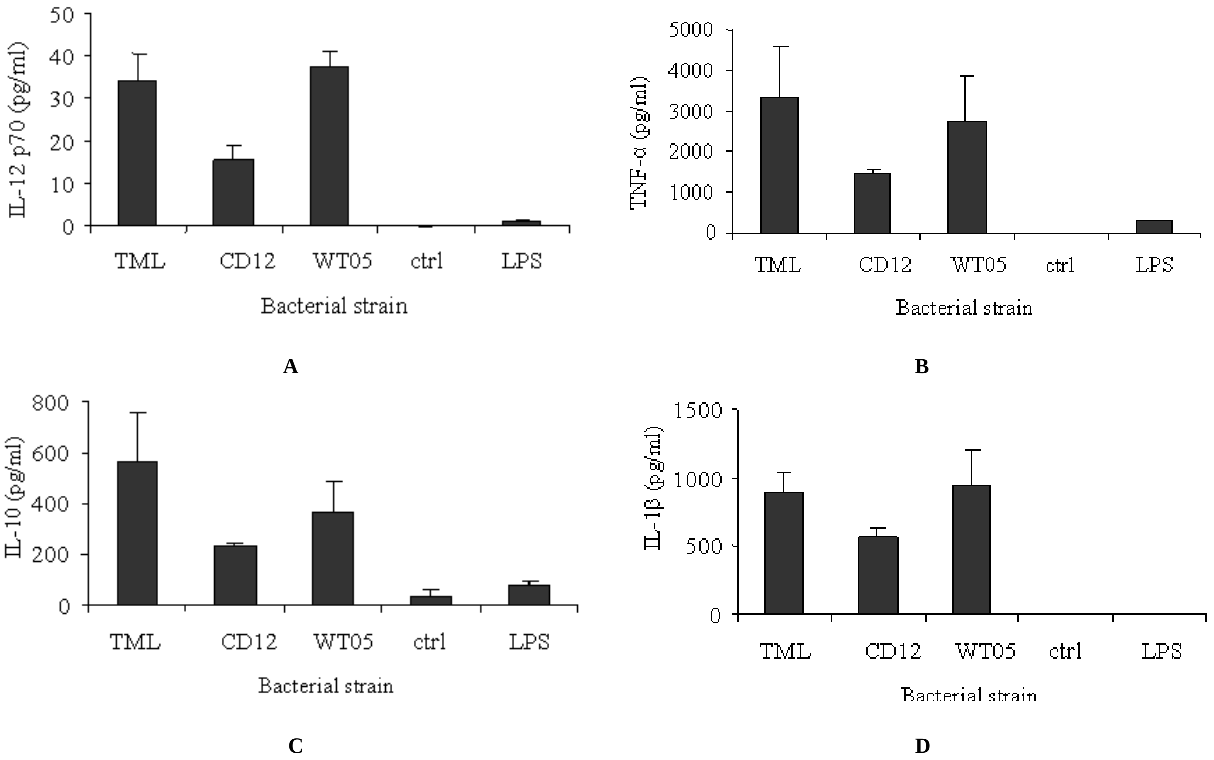

**FIGURE 2**. Graphs representing secretion of cytokines following infection of DC with strains CD12, WT05, and TML, as well as control (uninfected) DC, and DC after exposure to *S. typhimurium* LPS. Secretion of IL-12 p70 is shown in (A), TNF-α (B), IL-10 (C), and IL-1β (D). The MOI was 1:1 and the number of cells infected was $2 \times 10^5$. The difference between strains CD12 and WT05 was statistically significant for IL-12 release-*p* value 0.05 (as calculated by student T test); there was no statistical difference for TNF-α (*p* 0.46), IL-10 (*p* 0.46), IL-1β (*p* 0.36).

of EGFP to human cultured DC. CD12 and WT05 strains were evaluated as vectors for delivery of EGFP reporter gene to human cultured DC. Following infection of DC at the MOI 2:1 and 1:1, the cells were analysed by flow cytometry for EGFP expression. The *in vitro* gene transfer was successful, but the efficacy varied and was dependent on the MOI ratio and the viability of infected DC. The highest percentage of cells expressing EGFP, as determined by flow cytometry, was achieved for the strain WT05 (16.36%) at the MOI 2:1, and then CD12 (4.7%) at the MOI 2:1 (see Fig. 3).

### DISCUSSION

Attenuated *S. typhimurium* infection leads to maturation of DC and is subsequently capable of evoking a chain of immunological reactions. The interaction between DC is mediated by the TLR signalling that regulates three categories of signals that DC deliver to promote T-cell expansion: antigen, costimulation, and cytokines. The TLR involved in *S. typhimurium* infection is TLR-4. TLR-9, on the other hand, regulates activation induced by bacterial DNA CpG, forming yet another signal stimulating DC activation. Using attenuated *S. typhimurium* has potential advantages in therapy terms by not only transferring therapeutic genes to professional APC, the APC are themselves already activated to prime T cells. Attenuated *S. typhimurium* vaccine has been previously shown to be a more efficient way to vaccinate compared to antigen-loaded DC[13]. In the B16F1 melanoma model, the oral route of vaccination with attenuated *Salmonella* induced stronger expansion of cytotoxic T cells and helper T cells, as well as stronger tumour inhibition comparing to DC subcutaneous vaccine.

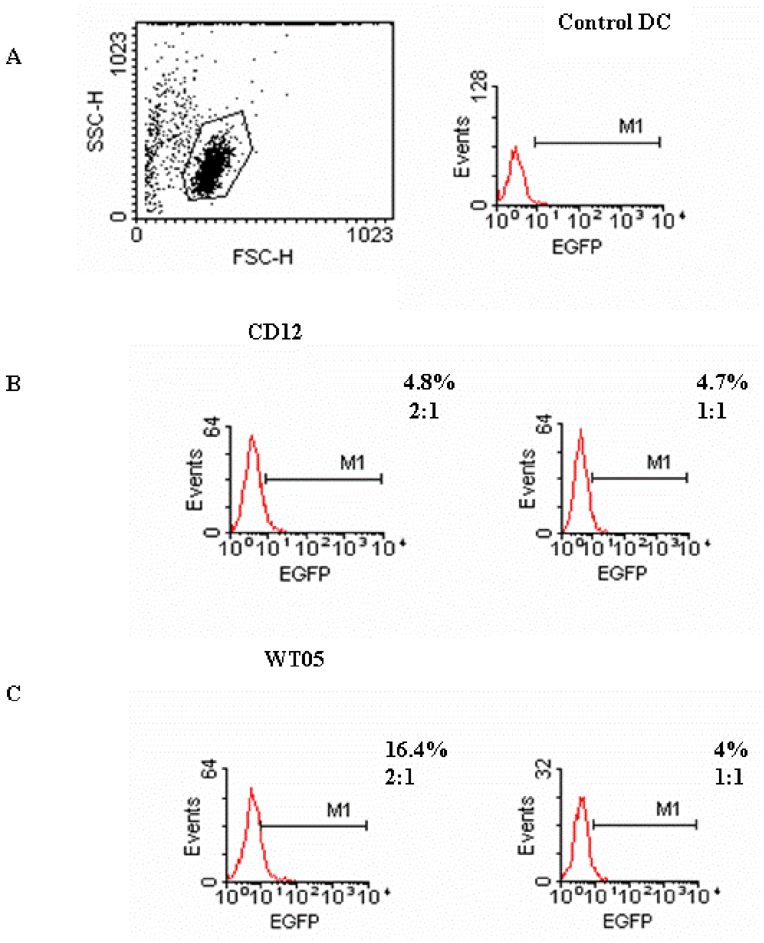

**FIGURE 3.** Graphs representing expression of EGFP in DC infected with *S. typhimurium* strains CD12 and WT05 as demonstrated by flow cytometry. Panel A shows the dot-plot graph with viable DC gated, as well as uninfected DC that were the negative control. Panel B represents EGFP expression by DC infected with CD12 at the MOI of 2:1 and 1:1, and panel C shows strain WT05. The figures above the histograms show the percentage of cells expressing EGFP as compared to negative control.

In this study, two new strains of *S. typhimurium* were assessed for invasiveness and gene transfer. Mutants with deletions in the *sifA* gene, such as CD12, are unable to maintain the integrity of their SVC[14,15] and may consequently prove to be more efficient at transferring DNA to the host than alternative strains. The novel strains of *S. typhimurium* CD12 and WT05 are capable of efficient maturation of DC as demonstrated by expression of maturation markers MHC I, MHC II, CD83, CD86, and CD40, as well as through cytokine release IL-12, TNF-α, IL-10, and IL-1β. Following infection, DC also secrete inflammatory chemokines CCL3, CCL4, and CCL5, which can contribute to skewing the inflammatory response towards Th1-type cellular response. Migration of DC to the sites of inflammation and then to draining lymph nodes is dependent on chemokine secretion as well as the expression of chemokine receptors. Chemokines are small secreted cytokines that act primarily as chemoattractants and activators of specific types of leukocytes. There is evidence that various types of chemokines are also involved in T-cell differentiation with some of them promoting Th1-type cellular response and others Th2[16], as reviewed in Chensue et al.[17]. DC express CCL3 (MIP-1α), CCL4 (MIP-1β), and CCL5 (RANTES), which may favour expansion of Th1 cells. A direct effect of CCL3 on T-cell differentiation was suggested by one study that showed that addition of CCL3 to antigen promoted development of IFN-

γ–producing cells[18]. *In vitro* supernatants from *S. typhimurium*–infected DC are chemoattractive to T cells and express macrophage-derived chemokine[19]. Polarising the immune response towards Th1-type cellular response is important in antitumour strategy.

The effect of different strains of *S. typhimurium* on maturation of DC was also reported by Dreher et al.[4] All types of attenuated bacteria matured DC, but secretion of IL-10 differed significantly, suggesting that certain mutants can suppress its release. The superior efficiency of DC maturation induced by live *S. typhimurium* compared to *S. typhimurium* LPS was reported by Pietila et al.[6].

The antigen presentation and *eukaryotic* protein expression in the context of *S. typhimurium*–delivered vaccine is an important issue that has been addressed in other studies. Flo et al. used attenuated *S. typhimurium* to vaccinate against Herpes simplex virus-2–induced disease[20]. By the introduction of an intron in the GFP, they have unequivocally demonstrated that the protein transcription was by *eukaryotic* nuclear process presented within MHC I. The protein was expressed within macrophages and DC, and elicited a strong cellular immune response in mucosal and systemic compartments. Similarly, orally administered DNA vaccine delivered within *Salmonella* was capable of inducing protective immunity against tumours in a murine melanoma model[21].

The data described, together with the fact that DC are capable of gene transfer as shown by EGFP expression, make them interesting candidates for gene delivery vehicles. Apart from carrying foreign genes, *Salmonella* also acts as a natural adjuvant in vaccine strategy due to LPS- and NK-induced activation, and potentates the response through acting on APC as described above[22]. Evidence from the literature also shows that *Salmonella* can target tumour cells. Pawelek et al. demonstrated that *S. typhimurium* can target multiple tumours from the distant inoculation site and it selectively replicates within tumours[23].

The demonstrated differences between two *S. typhimurium* strains have showed that one of them, WT05, is more suitable to be a vehicle for gene delivery. Strain WT05 matured DC in a more efficient way; caused higher release of inflammatory cytokines TNF-α, IL-12, and IL-1β; and was very efficient in gene transfer using EGFP as a reporter gene with 14% of infected DC expressing EGFP. Although the efficacy of *in vitro* gene transfer is still only in the range of 14%, it is much higher than in the other studies reported to date. Petrovska et al. reported the gene delivery studies with a *sifA* mutant and only approximately that 5% of live gated infected DC expressed the reporter EGFP gene[24]. These data are consistent with our studies of *sifA* mutant (CD12). The major limiting factor for assessment of bacterial activation of DC is their reduced viability and cell death that results from infection. This is likely to influence the levels of cytokine and chemokines, as well as efficacy of the gene transfer. The difference between the two strains is most likely caused by a genetic background of the attenuation, which may account for the different pattern of inflammatory immune response to *Salmonella* infection.

### ACKNOWLEDGEMENTS

We would like to thank The Special Trustees of St George's University of London and Cancer Vaccine Institute St George's University of London for their financial support.
